# Outcome and process evaluation of a social norms approach intervention on nonmedical use of prescription stimulants for study performance among Flemish university students: a quasi-experimental study

**DOI:** 10.1186/s13690-025-01603-6

**Published:** 2025-06-06

**Authors:** Katleen Derickx, Hanna van Roozendaal, Koen Ponnet, Benedicte Deforche, Annelies Thienpondt, Guido Van Hal

**Affiliations:** 1 Faculty of Medicine and Health Sciences, University of Antwerp, Wilrijk, Belgium; 2 Department of Communication Sciences, Faculty of Political & Social Sciences, Imec-Mict-Ghent University, Ghent, Belgium; 3 Department of Public Health and Primary Care, Faculty of Medicine and Health Sciences, Ghent University, Ghent, Belgium; 4 Faculty of Physical Education and Physiotherapy, Department of Movement and Sport Sciences, Vrije Universiteit Brussel, Brussels, Belgium

**Keywords:** Students, NMUPS, Misperceptions, SNA, Social media, Campaign, Health promotion, Belgium, Outcome assessment, Process assessment

## Abstract

**Background:**

Students are increasingly engaging in the nonmedical use of prescription stimulants (NMUPS) to enhance their study performance. However, little research has been conducted on the effectiveness of interventions to reduce NMUPS. This study assessed the effect of a social norms approach (SNA) intervention on NMUPS and the perception of NMUPS for study performance among Flemish students. Additionally, a process evaluation of the intervention was performed.

**Methods:**

A social media campaign, containing social norm messages based on data from ‘Head in the Clouds?’ (HITC) survey edition 2021, ran from December 2022 to April 2023 at the University of Antwerp. Data from the HITC survey was also used as baseline measurement (Antwerp: *n* = 2,963, Ghent: *n *= 8,598). Afterward, a post-intervention survey was conducted among the students of the University of Antwerp (*n* = 1,827) and Ghent University (*n* = 3,333), the latter serving as the control group. A quantitative process evaluation among the intervention group was conducted according to the guidance of the Medical Research Council for process evaluation of complex interventions.

**Results:**

A difference-in-difference approach showed that students of the intervention group at endline estimated NMUPS for study performance among peers significantly lower (*P* < .0001; Est. = -3.792; SE = 0.805)—and thus closer to the real social norm. There was no significant influence (*P* = 0.421; OR = 1.10; 95% CI = 0.87 to -1.39) of the intervention on NMUPS for study performance. The process analysis showed that 18.7% of the intervention group had seen the campaign. Most of them found the campaign credible (83.6%) and clear (website: 90.8%; videos 94.7%; images: 92.4%). The overall satisfaction was 6.38 (SD 1.68) out of 10. Very few students (3.1%) had seen the campaign via TikTok, compared to Facebook (64.0%) and Instagram (53.3%), although 35.7% of the total budget had been spent on TikTok.

**Conclusions:**

The results of this study confirm that an SNA intervention could reduce the misperceptions of NMUPS among students. However, the hypothesis that the behavior of NMUPS for study performance would be reduced subsequently could not be demonstrated. Follow-up research is needed to investigate long-term effects. The implementation of the intervention might be improved by making more optimal use of the campaign budget.

**Supplementary Information:**

The online version contains supplementary material available at 10.1186/s13690-025-01603-6.

**Table:** 

Text box 1. Contributions to literature
• The difference between students’ perceptions of their peers’ nonmedical use of prescription stimulants (NMUPS) for study performance and the actual level of NMUPS for study performance among Flemish students suggests that a social norms approach (SNA) intervention could be beneficial.
• This study is the first in Belgium to implement and assess an SNA intervention aimed at addressing NMUPS for study performance and the misperceptions surrounding peers’ NMUPS for study performance among university students.
• The findings from this study demonstrate the effectiveness of an SNA campaign in correcting students’ misperceptions about NMUPS for study performance.
• Insights from the process evaluation might help other universities create similar SNA campaigns for their students.

## Background

Prescription stimulants, such as methylphenidate, modafinil, and (dextro)amphetamine, are increasingly being inappropriately used among students to improve their study performance [1, 2]. Inappropriate use refers to NMUPS, i.e., in the absence of a diagnosis of attention deficit hyperactivity disorder (ADHD) or narcolepsy [3]. In Europe, the overall point prevalence of NMUPS for study performance varies between 0.8% and 16% [4]. The highest prevalences occur among young adults up to 25 years old [2]. Among Flemish higher education students, 7.0% reported in 2021 having used at least once prescription stimulants to improve their academic performance, and 4.4% over the past twelve months, with a peak during exam periods [5].

Besides ethical considerations regarding equal opportunities, fairness, and academic integrity, NMUPS for enhancing study performance also comes with potential health risks. Side effects associated with stimulant use include headache, stomachache, reduced appetite, increased blood pressure, panic attacks, blackouts, disruption of sleep patterns, and—in the long term—mental health problems and drug addiction [2, 6, 7]. About eight out of ten Flemish students who have ever used stimulant medication to improve their study performance reported experiencing at least one negative side effect [5]. Moreover, it is known that unhealthy and risky behavior for other substances, such as alcohol use, often originates during student life and may persist into adulthood [5, 8–10].

When studying students’ behavior, their social context should be taken into account [11–15]. Health behavior is influenced by the social norms of the reference network to which individuals belong [16, 17]. In addition, people often overestimate the prevalence of unhealthy behavior among their peers. These misperceptions can lead to unhealthier behavior because they are falsely perceived as the norm that one should emulate. An overestimation of NMUPS among peers has also been observed among European students [18]. Prior research suggests that a social norms approach (SNA) could be a successful prevention strategy [3, 19]. The goal of SNA is to correct the overestimated perceptions of unhealthy peer behavior [20]. While little post-intervention research has been conducted on the effectiveness of interventions for NMUPS [2], the SNA approach has proven effective in other health behavior contexts, such as unhealthy alcohol consumption [14, 21].

Based on the conceptual framework by Keller and Bauerle [22], it is hypothesized that the SNA will reduce the misperceptions of NMUPS for study performance, and subsequently decrease NMUPS. Originally, the model was designed for unhealthy drinking behavior. Analogous to this model, a beneficial effect of an SNA intervention for NMUPS could be expected when the underlying problem satisfies the three conditions of the model; namely, the conditions of the prevalence of NMUPS for study performance, the prevalence of misperceptions, and an association between both. The framework suggests that correcting the misperception of NMUPS for study performance is necessary before the behavior itself declines [23].

SNAs have proven to be a promising and cost-effective method for changing behavior when conducted according to best practices [14]. However, previous research has often overlooked using a pre-intervention baseline measurement from the target group, and/or including a comparison group. Additionally, maintaining methodological rigor and clear reporting has proven challenging [24].

For the implementation of the intervention, it is recommended to use innovative approaches to engage the target participants, like the use of social media [24]. Research has shown that social media channels, due to their widespread use among higher education students, can significantly enhance the reach and impact of public health campaigns [25–27]. The practical design and the implementation process of the SNA intervention would benefit from using additional evaluation tools, for example, qualitative studies on participant experiences and/or process evaluations, which provide a greater understanding of the underlying mechanism of the intervention effects [14, 28].

Taking into account the aforementioned concerns and pitfalls, this study aimed to assess the effect of an SNA intervention on NMUPS for study performance among university students. The secondary aim was to assess the anticipated intermediate effect of the SNA on the students’ perceptions of NMUPS for study performance among their peers. The specific application of the SNA is elaborated in more detail in the subsequent sections. Additionally, the process of the intervention was investigated.

## Methods

This research on NMUPS among Flemish students formed part of a larger study in which a similar campaign on alcohol use among students was set up. The study was a collaboration between the University of Antwerp, the City of Antwerp, Ghent University, and the Flemish expertise center on alcohol and other drugs (VAD) [29].

### Study design

A quasi-experimental study design was used to assess the effect of an SNA intervention on the (perception of) NMUPS among Flemish university students. The assignment to the intervention was nonrandomized; students of the University of Antwerp were exposed to the intervention, and students of Ghent University were selected as the control group. The intervention was set up as a social media campaign and ran between December 2022 and April 2023.

The ‘Head In The Clouds?’ (HITC) survey edition 2021 [5] served as a baseline measurement. The HITC survey concerns a quadrennial student survey on substance use among students at all Flemish universities and colleges of higher education, and is a collaboration between VAD and the Flemish higher education institutions. After the intervention, a post-intervention survey was conducted among the university students, in both the intervention and control groups. In both surveys, convenience sampling was used.

For the design and reporting of the study, the Transparent Reporting of Evaluations with Nonrandomized Designs (TREND) statement was followed [see Additional file 1]. The TREND statement provides a guideline for enhancing transparency and quality in reporting [30].

### Study setting and population

The study population consisted of students from 17 to 25 years of age. This age range corresponds to the period when students enter higher education and begin their professional careers. The focus is on this target group—as they are more prone to risky behavior due to academic demands and pressures—in order to achieve the maximum impact of the SNA campaign.

The study involved two Flemish universities in Belgium; the University of Antwerp and Ghent University. These universities are representative of other universities in Belgium and Europe regarding the outcome variable of NMUPS for study performance. At baseline, the overall NMUPS to enhance study performance among Flemish students over the past twelve months was 4.4%, which is in line with NMUPS rates in Wallonia (the southern part of Belgium) and other European countries [12, 31].

The students of the University of Antwerp were exposed to an SNA intervention to promote healthy behavior, and students of Ghent University served as the control group. Students of Ghent University who answered in the post-intervention survey that they had seen the campaign were excluded from the study.

### Intervention

#### Development of SNA intervention

The SNA intervention consisted of a campaign using social norm messages disseminated to students through social media. For the development of accurate social norm messages on NMUPS for study enhancement, data from the HITC survey ed. 2021 from the students of the University of Antwerp were used (*n* = 2,963). These students were recruited through a convenience sampling method. After excluding respondents older than 25 years (*n* = 243) and those who did not complete the questions on social norms and NMUPS (*n* = 831), 1,941 students remained. No weighting procedures were applied to this data for the development of the social norm messages.

Of these students, 6.8% answered “yes” to the question “Have you ever used stimulant medication to improve your study performance?”. Notwithstanding, students think that on average 36.68% (CI: 35.80% – 37.56%) of students have ever used prescription stimulants to enhance their study performance. The question in the survey was: “What percentage of students do you think have ever used stimulant medication to improve their study performance?”. Here, a clear difference between the actual norm and the perceived norm can be observed. Other questions probed NMUPS to improve study performance in the past twelve months (4.7%), in the past twelve months during class periods (4.1%), and in the past twelve months during exam periods (6.5%).

Based on these answers, we formulated the following social norm messages:

- 93% of students have never used stimulant medication to improve their study performance.
- In the past 12 months, more than 95% of students have not used stimulant medication to improve their study performance.
- During exam periods, almost 94% of students never use stimulant medication to improve their study performance.
- During lecture periods, even 96% of students never use stimulant medication to improve their study performance.

Following the guidelines of the National Social Norms Center at Michigan State University (US) [32, 33], efforts were made to formulate the messages credible, powerful, stressing positive behavior, and focused on encouraging healthy behavior.

#### Social media campaign

**Fig. 1:**
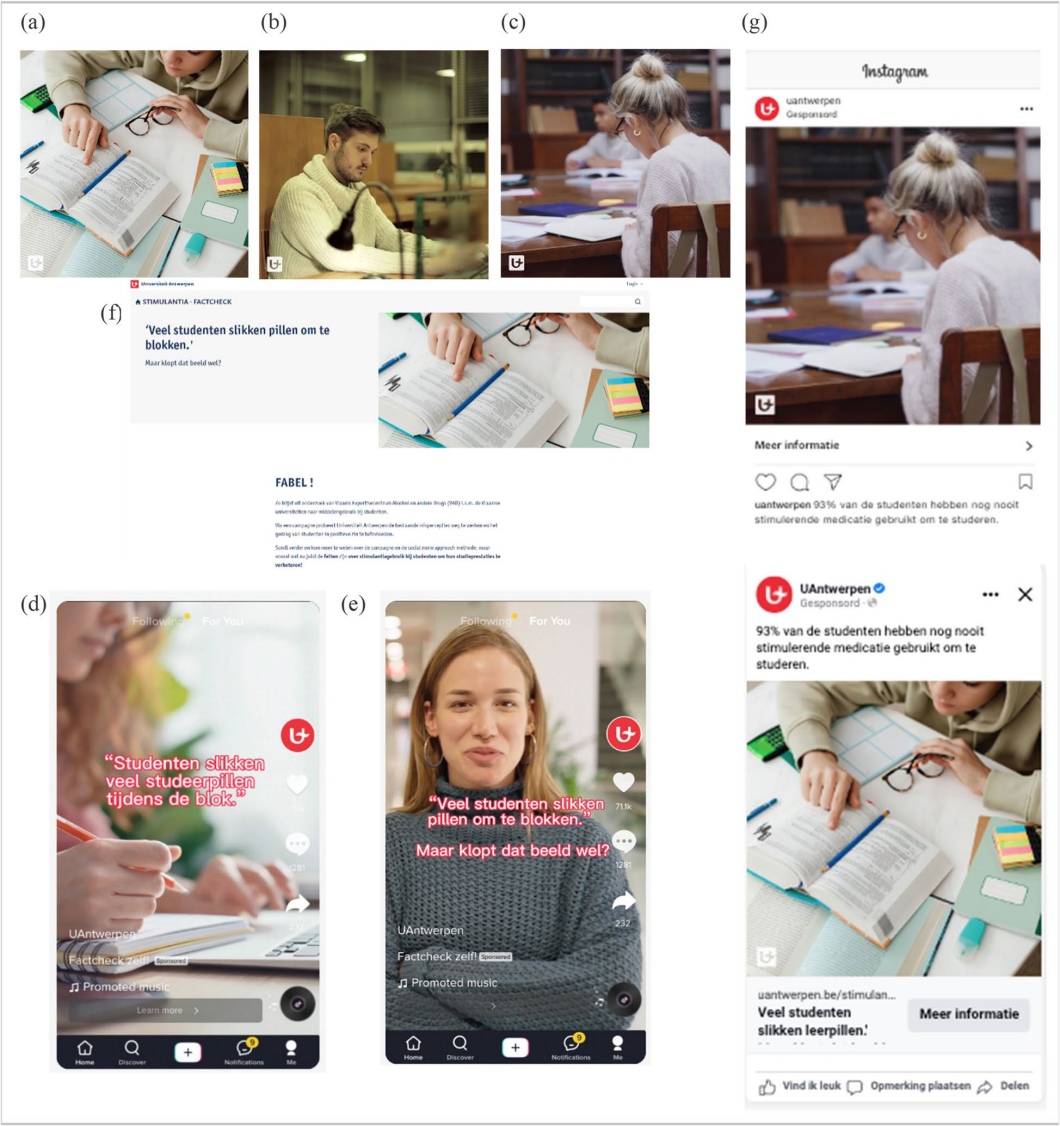
Social Norms Approach campaign on nonmedical prescription stimulant use among Flemish university students in 2022–2023

Based on these messages, the University of Antwerp's communications department was asked to set up a social media campaign. The campaign material included three static images, each featuring one of the four social norm messages (Fig. 1a, b, c, g), along with two short videos (Fig. 1d, e) and a website [34] (Fig. 1f). The website featured the campaign materials and provided additional information about the study and the social norms approach theory. It also included references to other informative websites about NMUPS.

The NMUPS campaign was launched on December 14 th, 2022, and ran initially for a period of five weeks. The University of Antwerp’s communications department posted paid advertisements through the social media channels TikTok, Facebook, and Instagram. The objective of this initial campaign was set to refer the viewers to the website. The target audience for TikTok was set to 18- to 25-year-olds, originating from Belgium, and interested in university, college education, students, and/or student life. 13- to 17-year-olds were mistakenly included in the target audience. The target population for Meta (Facebook and Instagram) were 18- to 25-year-olds, originating from Flanders, Ghent excluded, and interested in university. The budget for TikTok was set at a maximum of €200, and the budget for Meta was a maximum of €20 per day. The campaign ran on TikTok from December 14 th to December 28 th, 2022, and on Meta from December 14 th, 2022, to January 20 th, 2023. Besides the paid advertisements, student associations were asked to share the campaign on their social media channels, and, through the website, students could also spread the campaign within their own networks.

The boost campaign ran from March 31 st, 2023, to April 16 th, 2023, and aimed to refresh the initial campaign. The execution and the budget set of the campaign were similar to those of the initial campaign, except that the 13- to 17-year-olds were excluded from the target audience on TikTok.

Although there is no evidence of the ideal duration time for an SNA campaign, it was consulted with the study team to run the intervention for 18 weeks. This 18-week duration allowed for questioning both the lecture and exam periods in the post-intervention measurement.

### Data

#### Data collection

Data from the HITC survey edition 2021 of the students from the University of Antwerp and Ghent University served as baseline measurement. The endline measurement consisted of a post-intervention survey among the students of the University of Antwerp (intervention group) and students of Ghent University (control group). The survey took place between April 17 th and May 11 th, 2023. The students were recruited for the post-intervention survey by email and the student portals, similar to the recruitment for the HITC survey in 2021. The surveys were anonymous, and informed consent had been obtained from each student who participated in the post-intervention survey. The post-intervention survey was created using Qualtrics® version 2022 software [35].

#### Outcomes

The primary outcome variable was the change in NMUPS to enhance study performance among students. Similar to the HITC ed. 2021 baseline survey, this was assessed using the binary (yes/no) item “In the past 12 months, did you use prescription stimulants to improve your study performance?”. The secondary outcome was change in students’ perception of other students’ NMUPS to enhance study performance. This was, similar to the HITC survey ed. 2021, questioned by the item “What percentage of students do you think have ever used prescription stimulants to enhance their study performance?”.

In addition to evaluating the intervention's effects, a process evaluation was conducted to better understand how the intervention worked. The framework used for this evaluation was the Medical Research Council (MRC) guidance framework for process evaluations of complex interventions [28]. This choice was made because the campaign was a university-wide intervention targeting students in a context characterized by interaction and interdependence. Insight was gained into the implementation process, the mechanisms of impact (level of satisfaction, perceived benefit, and relevance), and the contextual factors involved. The process evaluation was questioned by means of statements and questions for the different parts of the campaign (images, videos, and website) and the campaign as a whole [see Additional file 2]. The overall perceived level of satisfaction about the intervention, for example, was assessed using the item “What do you think of the student campaign on stimulant medication as a whole?” with a 0 to 10 numerical rating scale where 0 was very poor and 10 was very good. Data regarding the spent budget, reach of the campaign, number of impressions, and number of clicks to the website were provided by the communication department of the University of Antwerp. These metrics were obtained through Google Analytics and Meta Insights.

#### Covariate data

Directed acyclic graphs (DAGs) were drawn a priori to identify covariates that possibly impact the intervention; one for the relationship between the SNA intervention and NMUPS for study enhancement among students, and a second DAG for the relationship between the SNA intervention and the perception of NMUPS for study enhancement among peers [see Additional file 3].

The selection of potential confounders was based on the data of the baseline measurement, literature review, and expert opinions. The identified possible confounding variables included the characteristics birth sex (male/female), age, faculty at which the student was studying, occupation (working student: yes/no), type of current education (bridging program/bachelor/master/other), living situation on weekdays (at parental home/independently), active fraternity member (yes/no), and religion. Other identified possible confounders were other substance use, life satisfaction, mental distress, and academic stress. These items were repeated from the HITC ed. 2021 survey in the post-intervention questionnaire. Some variables that could be confounding variables, but were not available in the HITC ed. 2021 survey, were added to the post-intervention survey. For example, subjective norm, previous exposure to other campaigns, procrastination, and perfectionism.

##### Other substance use

Students were questioned binary (yes/no) on tobacco, tranquilizers, and cannabis use in the past twelve months, and ever-use of other illegal drugs. Alcohol consumption was questioned using the 3-item Alcohol Use Disorders Identification Test (AUDIT-C) questionnaire, with a total score on a scale from 0 to 12. AUDIT-C is a screening test for heavy drinking and/or alcohol abuse or dependence [36].

##### Mental well-being

Life satisfaction was questioned using the Cantril ladder, with steps from 0, representing the worst possible life, to 10, representing the best possible life [37]. Mental distress was surveyed by the Kessler- 6 psychological distress scale [38] (Cronbach’s α = 0.86). Academic stress was questioned using the 11-item College Student Stress Scale (CSSS- 11) [39] (Cronbach’s α = 0.88). Both scales were scored along a 5-point Likert scale, ranging from 1 = never to 5 = very often for CSSS- 11, and from 0 = never to 4 = very often for the 6-item Kessler- 6 scale.

##### Subjective norm

Refers to the perceived social pressure to perform or not perform a particular behavior. The student’s estimate of the social pressure to engage in the target behavior was measured by two items: “People who are important to me would approve of me taking stimulants to enhance my study performance” and “People expect me to take stimulants if it would improve my study performance”. The items were scored using a 5-point Likert scale, ranging from 1 = strongly disagree to 5 = strongly agree [13].

##### Procrastination

Levels of procrastination were measured using a validated measurement instrument; the Tuckman’s procrastination scale [40]. This scale contains 16 items and is scored along a 4-point Likert scale (Cronbach’s α = 0.71), ranging from 1 = That’s not me for sure to 4 = That’s me for sure.

##### Perfectionism

The 8-item short form of the revised almost perfect scale (SAPS) with a 7-point Likert scale was used to measure the level of perfectionism [41, 42] (Cronbach’s α = 0.86), ranging from 1 = strongly disagree to 7 = strongly agree.

#### Sample size

Due to a lack of previous research on the effect of an SNA intervention on NMUPS, a small effect size of 0.15 was assumed. Taking into account a significance level of 0.05 (two-sided test), and a power of at least 80%, a minimum of 699 subjects in the intervention group and 699 subjects in the control group were required to participate in the study. These calculations did not take into account the possible effects of clustering within study trajectories and/or faculties. G*Power 3.1.9.4 software was used for the sample size calculation.

#### Statistical analysis

A difference-in-differences (DiD) approach was applied to measure the impact of the intervention on the outcome variables. This approach, where the average change over time of an outcome variable is compared between an intervention and control group, is commonly used for quasi-experimental research to reduce bias when certain groups are more likely to receive the intervention than others because of characteristics related to the outcome [43]. In addition, a linear mixed effects model was used for the analysis of the perceived social norm of NMUPS among peers, and a generalized linear mixed model (GLMM) was used for the analysis of NMUPS. These models take into account dependency due to possible clustering effects within faculties.

Missing values were imputed using multiple imputation (MI) [44]. MI assumes that the missing values are missing at random (MAR). Data on missingness can be found in the supplementary material [see Additional file 4]. By applying multiple imputation with chained equations (using the mice package in R [45, 46]), ten independent datasets were generated, both for intervention and control groups, and base- and endline.

Since the intervention was not allocated at random, inverse probability weighting (IPW) was applied to control for differences between baseline groups and endline groups [see Additional file 5] [47–49]. Based on observable characteristics, individuals were assigned a propensity score, i.e., the probability that an individual would participate in the intervention group. Based on the propensity scores, a weight for each individual had been calculated. IPW was applied separately to the baseline and endline datasets, and for the different imputed datasets. Final data analyses were performed on the ten independent datasets, and from there, an inferential analysis was pooled.

The process evaluation was done according to the Medical Research Council guidance for process evaluation of complex interventions [28, 50], which gave insight into the implementation process of the intervention, the mechanisms of impact, and the context, in order to better understand how the intervention worked.

All statistical analyses were performed using RStudio® version 2021.09.0 [46].

## Results

### Participant flow

**Fig. 2:**
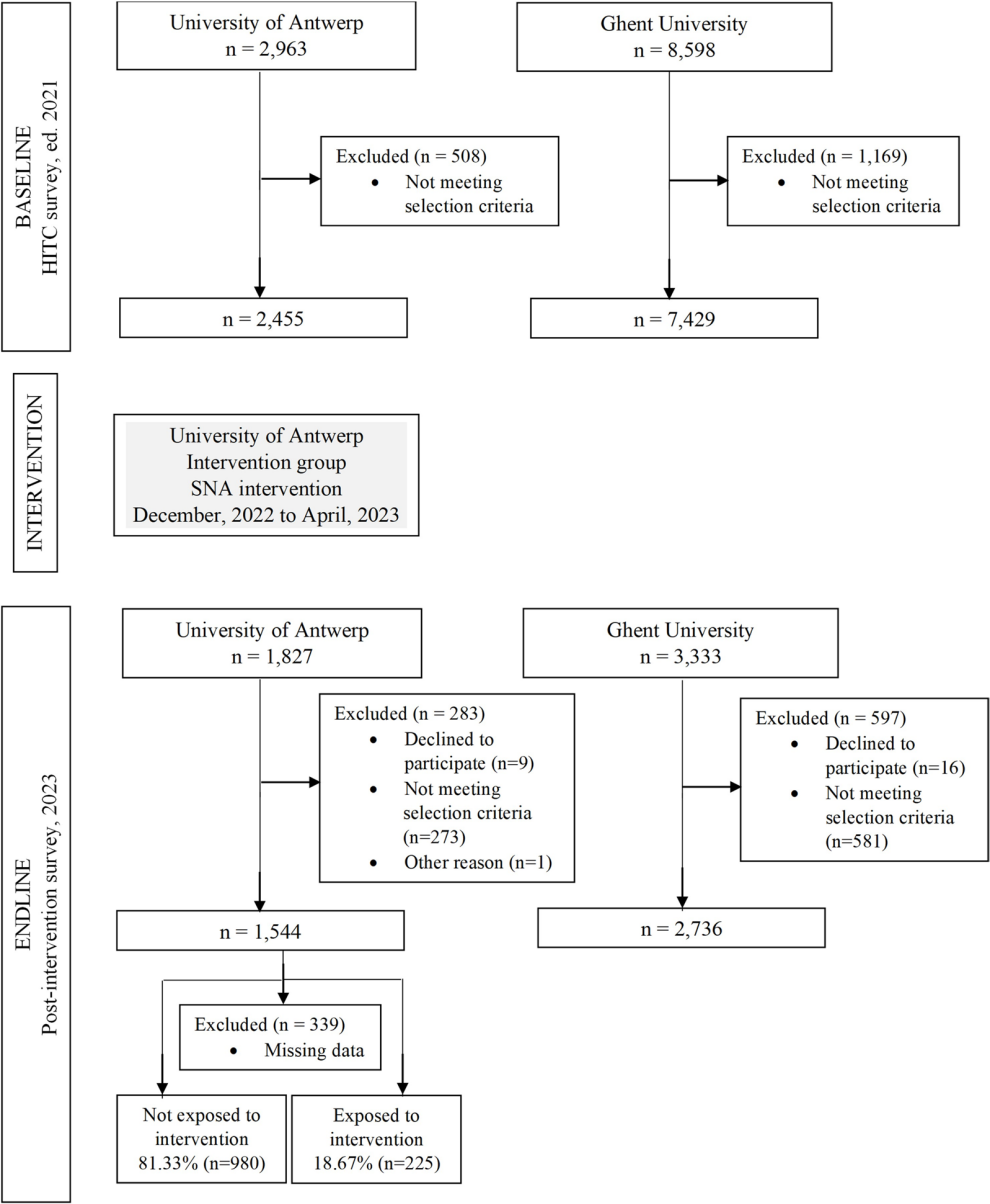
Flow diagram of an SNA intervention on NMUPS for study performance among Flemish university students

At baseline, 2,455 eligible students from the University of Antwerp and 7,429 eligible students from Ghent University completed the HITC survey (ed. 2021). At endline, 1,544 eligible students from the University of Antwerp and 2,735 eligible students from Ghent University filled out the post-intervention questionnaire. Students from Ghent University who had been exposed to the campaign were excluded from the control group (*n* = 55). Additionally, one student from Antwerp who clearly had not completed the post-intervention questionnaire seriously was excluded. A flow diagram of the survey participants is shown in Fig. 2.

### Characteristics of study participants

Table 1 shows the distribution of students from the University of Antwerp (intervention group) and Ghent University (control group) across variables. Both at baseline and endline, the majority of respondents were female: 61.98% (6126/9884) and 60.79% (2601/4279) respectively. The mean age was similar across all groups. Differences between the intervention and control groups were noticed in the distributions of faculties, type of education, living situation on weekdays, religion, and being an active fraternity member. IPW was used in the analysis to adjust for these differences.

**Table 1:** Students’ characteristics at Baseline (2021) and Endline (2023)

	**Baseline** *n* = *9884*		**Endline** *n* = *4279*	
	**Intervention group**	**Control group**	**Intervention group**	**Control group**
	*n* = *2455*	*n* = *7429*	*n* = *1544*	*n* = *2736*
**Sex, n(%)**				
Female	1500 (61.2)	4626 (62.3)	925 (59.9)	1677 (61.3)
Male	952 (38.8)	2803 (37.7)	618 (40.1)	1057 (38.7)
**Age (years)**, median [IQR]	21.0 (4.0)	21.0 (3.0)	20.0 (3.0)	21.0 (3.0)
**Faculty, n(%)**				
Medicine and health sci	377 (15.4)	1299 (17.5)	249 (16.1)	494 (18.1)
Veterinary and pharm. sci	362 (14.8)	502 (6.77)	242 (15.7)	176 (6.44)
Engineering sciences	384 (15.7)	1455 (19.6)	232 (15.0)	465 (17.0)
Exact sciences	271 (11.0)	518 (6.99)	211 (13.7)	228 (8.34)
Economics	309(12.6)	915 (12.3)	146 (9.46)	270 (9.87)
Social sci. and psychology	226 (9.21)	1,371 (18.5)	115 (7.45)	541 (19.8)
Linguistics and philosophy	300 (12.2)	803 (10.8)	217 (14.1)	367 (13.4)
Law and criminology	224 (9.13)	547 (7.38)	131 (8.49)	194 (7.09)
**Education, n(%)**				
Bridging program	126 (5.13)	408 (5.49)	82 (5.31)	138 (5.05)
Bachelor’s program	1556 (63.4)	4143 (55.8)	1074 (69.6)	1625 (59.4)
Master’s program	717 (29.2)	2,642 (35.6)	368 (23.8)	883 (32.3)
Other	56 (2.28)	236 (3.18)	19 (1.23)	88 (3.22)
**Living situation weekdays, n(%)**				
At parental home	1575 (64.2)	2,976 (40.1)	926 (60.9)	985 (36.6)
Independently	878 (35.8)	4451 (59.9)	594 (39.1)	1703 (63.44)
**Working status, n(%)**				
Not working	1712 (70.2)	5633 (76.5)	925 (60.0)	1832 (67.1)
< 20 h/week	648 (26.6)	1474 (20.0)	553 (35.9)	797 (29.2)
> 20 h/week	78 (3.20)	258 (3.50)	64 (4.15)	100 (3.66)
**Religion, n(%)**				
Christian	784 (32.0)	2777 (37.4)	373 (24.4)	846 (31.4)
Jewish	9 (0.37)	3 (0.04)	3 (0.20)	4 (0.15)
Islamic	94 (3.83)	143 (1.93)	59 (3.87)	36 (1.34)
Hindu	5 (0.20)	12 (0.16)	2 (0.13)	5 (0.19)
Buddhist	5 (0.20)	32 (0.43)	3 (0.20)	7 (0.26)
No religion	1456 (59.4)	4226 (56.9)	1024 (67.1)	1705 (63.4)
Other	99 (4.04)	229 (3.09)	62 (4.06)	87 (3.23)
**Active fraternity member, n(%)**				
Yes	275 (14.7)	826 (12.6)	310 (20.6)	430 (16.3)
**Social norm**, mean (SD)	36.7 (19.8)	36.2 (19.2)	33.4 (19.5)	36.0 (21.4)
**Past-year NMUPS, n(%)**				
Yes	88 (4.61)	263 (4.03)	78 (6.59)	101 (5.11)
**Past-year Tobacco use, n(%)**				
Yes	585 (24.1)	1803 (24.4)	362 (31.8)	535 (29.3)
**AUDIT-C score**, median [IQR]	2.0 [3.0]	3.0 [3.0]	3.0 [6.0]	3.0 [5.0]
**Past-year tranquilizer use, n(%)**				
Yes	64 (3.58)	175 (2.84)	86 (7.56)	123 (6.74)
**Past-year cannabis use, n(%)**				
Yes	456 (23.5)	1804 (27.0)	290 (25.5)	464 (25.5)
**Ever-use illegal drugs, n(%)**				
Yes	275 (14.2)	824 (12.4)	177 (15.5)	255 (14.0)
**CSSS scale**, median [IQR]	30.0 [12.0]	30.0 [11.0]	29.0 [12.0]	29.0 [11.0]
**Kessler scale**, median [IQR]	11.0 [8.0]	11.0 [8.0]	9.0 [7.0]	9.0 [7.0]
**Cantril scale**, median [IQR]	6.0 [2.0]	6.0 [2.0]	7.0 [2.0]	7.0 [2.0]
**Procrastination**, median [IQR]	-	-	40.0 [8.0]	40.0 [7.0]
**Perfectionism**, median [IQR]	-	-	36.0 [12]	36.0 [12.0]
**Subjective norms**, median [IQR]	-	-	3.0 [3.0]	3.0 [2.0]
**Exposure to other campaigns, n(%)**				
Yes	-	-	3 (0.25)	7 (0.35)

### Outcome evaluation

#### NMUPS for study performance

**Fig. 3:**
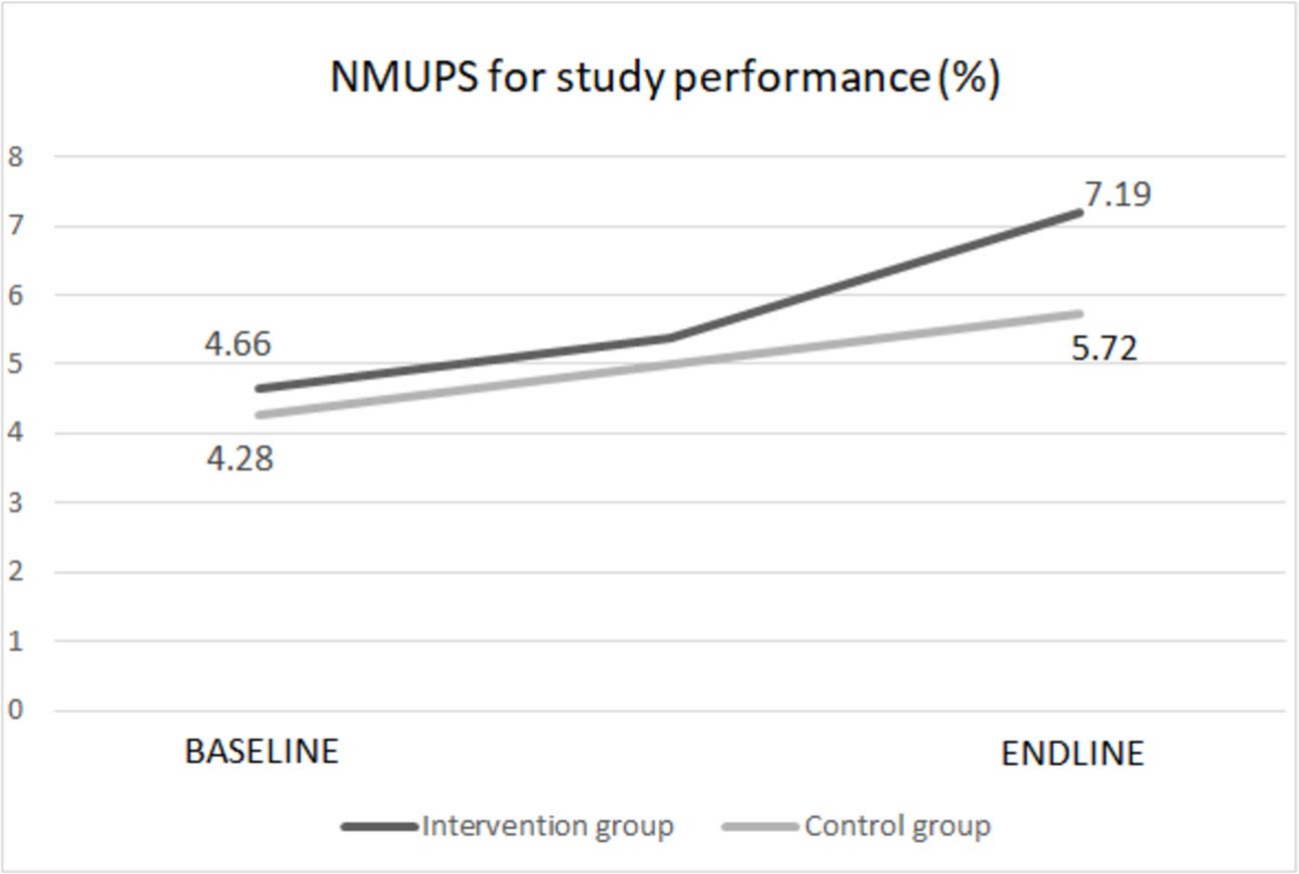
Effect of SNA campaign on NMUPS for study performance among Flemish university students in 2022–2023

At baseline, 4.66% of the students of the University of Antwerp and 4.28% of the students of Ghent University used non-medically prescription stimulants in the past twelve months to enhance their study performances. At endline, 7.19% of the students of the University of Antwerp and 5.72% of the students of Ghent University used these stimulants to improve their study performances (Fig. 3). Among the students at the University of Antwerp who had seen the campaign, 7.03% used non-medically prescription stimulants to improve study performance. The percentages provided represent weighted averages, calculated using IPW.

A generalized linear mixed model was employed to predict the effect of the SNA intervention on NMUPS for study performance. Table 2 shows that there was no significant influence (*P* = 0.421, OR = 1.10; CI = 0.87–1.39) of the intervention on NMUPS for study performance. Since there could be some dependence within faculties, faculty was included in the model as a random effect. The estimated variance of the random effect was 0.0019, which indicates minimal variance between the faculty groups.

**Table 2:** Effect of intervention on NMUPS for study performance

**Fixed effects**				
	Est	SE	*p*	OR (95% CI)
Intercept	− 3.109	0.078	<.0001	
Time				
Endline	0.347	0.088	0.0003	1.42 (1.19–1.68)
Group				
Intervention	0.111	0.069	0.186	1.12 (0.98–1.28)
Time*group				
Endline*intervention	0.093	0.119	0.421	1.10 (0.87–1.39)
**Random effect**				
	Est	Std. dev	N group	
Faculty (intercept)	0.0019	0.162	8	

#### Perceived social norms of NMUPS for study performance

**Fig. 4:**
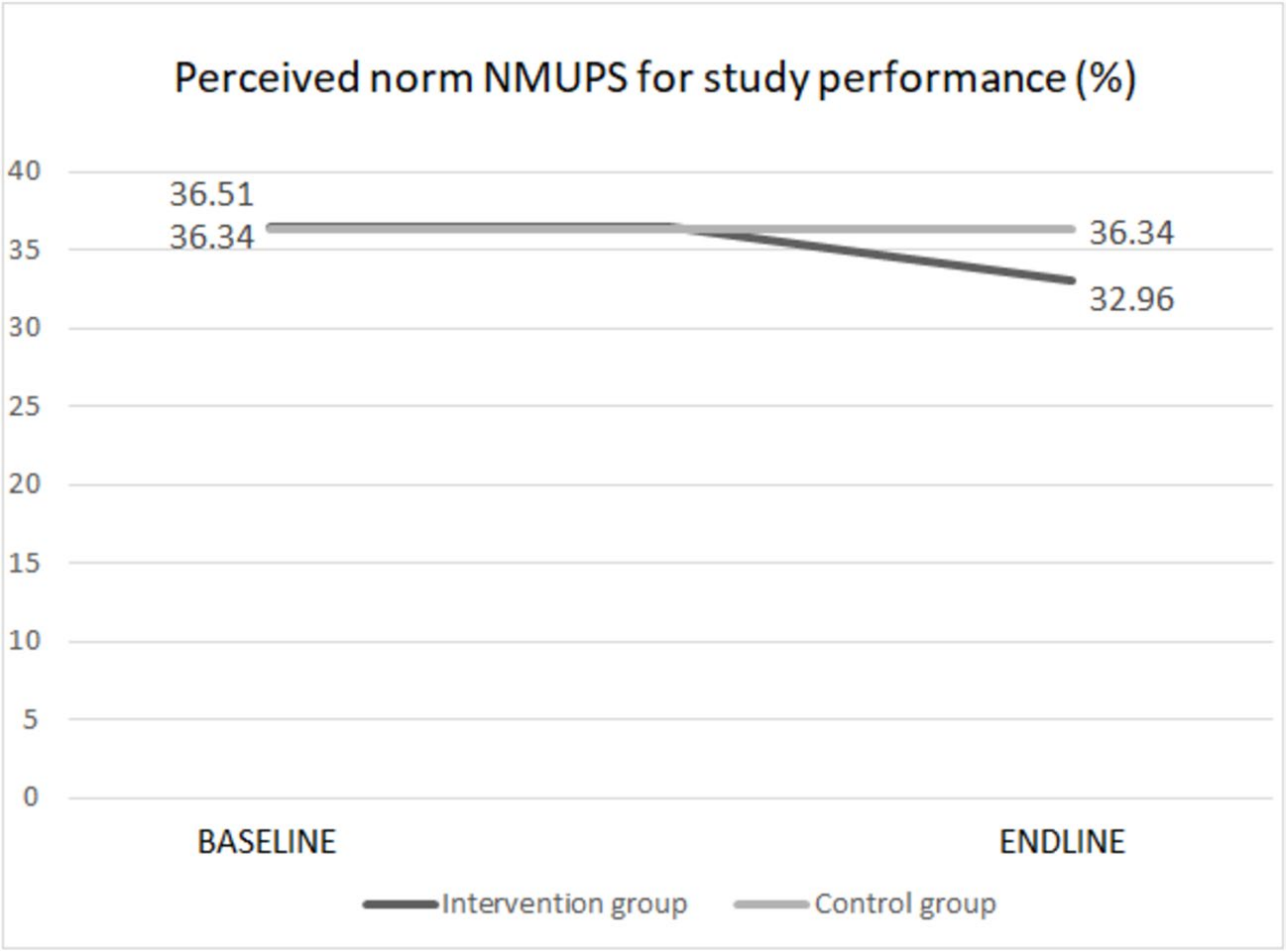
Effect of SNA campaign on perceived social norm of NMUPS for study performance

At baseline, students at the University of Antwerp and Ghent University thought on average that 36.51% and 36.34% of the students respectively use prescription stimulants to enhance their study performances. At endline, this perception decreased to 32.96% in the intervention group and remained stable at 36.34% in the control group. A relative intervention effect of 9.72% decrease was observed (Fig. 4). Among those who actually saw the campaign, an even larger relative intervention effect of 15.42% decrease could be observed; the perception of NMUPS for study enhancement decreased to 30.88%. These results are weighted means.

Results of a linear mixed effects model revealed that students of the intervention group at endline estimated the NMUPS for study performance significantly lower (*P* < 0.0001; Est. = − 3.792; SE = 0.805)—and thus closer to the real social norm—compared to the students of the control group (Table 3). The estimated variance of the random effect is very low (6.528 10^–13^), which implicates a minimal variance between the different faculty groups.

**Table 3:** Effect of intervention on perceived social norm of NMUPS for study performance

**Fixed effects**			
	Est	SE	*p*
Intercept	36.467	0.794	<.0001
Time			
Endline	0.113	0.559	0.840
Group			
Intervention	0.174	0.458	0.705
Time*group			
Endline*intervention	− 3.792	0.805	<.0001
**Random effect**			
	Est	Std. dev	N group
Faculty (intercept)	6.528 10^–13^	2.012	8

A post-hoc exploratory subgroup analysis of male and female students showed that the intervention was effective for both males and females, but the effect was stronger for women [see Additional file 6].

### Process evaluation

#### Implementation

As planned, the total amount spent on TikTok for the initial campaign was €200. In total, there were 168,766 impressions and 828 clicks to the website, equivalent to a cost per click of €0.24. A detailed analysis of age and sex showed that €61.22 (30.6%) of the total amount went to 13- to 17-year-old females, €67.22 (33.6%) to 18- to 24-year-old females, €31.75 (15.9%) to 13- to 17-year-old males, and €39.81 (19.9%) to 18- to 24-year-old males. There were 48,066 impressions on TikTok for 13- to 17-year-old females, 57,404 for 18- to 24-year-old females, 30,477 for 13- to 17-year-old males, and 32,819 for 18- to 24-year-old males, with respectively 258, 265, 142, and 163 clicks. The Click-Through-Rate (CTR) comes to respectively 0.54%, 0.46%, 0.50%, and 0.47%.

**Fig. 5:**
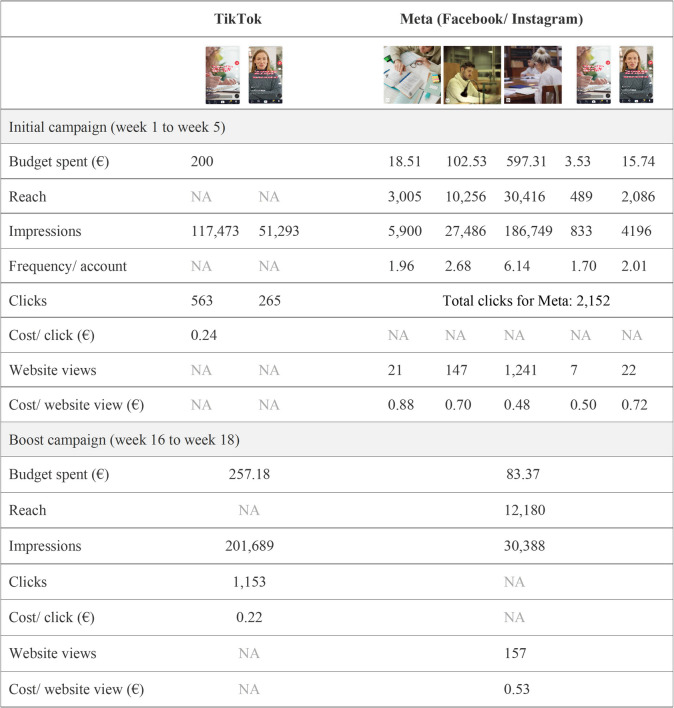
Detail of process evaluation metrics for the different components of the campaign, NA = data is not available

For Meta, the total amount spent for the initial campaign was €739.50. There was a total reach of 30,832 unique individuals, an average frequency of displayed content per unique individual of 7.32, and a total of 225,639 impressions. Of the 2,152 clicks, there were 1,442 views of the website. The average cost per view of the website was €0.51. Among the individuals who clicked through to the website, 38% (546/1442) were male and 62% (895/1442) female, corresponding to a click-through-cost of respectively €0.54 and €0.49. The static images (Fig. 1a, b, c) reached more individuals and had more views than the videos (Fig. 1d, e). Figure 5 shows a more detailed overview of the metrics obtained through Google Analytics and Meta Insights.

Some minor adaptations were implemented during the boost campaign. Because it turned out that, in the course of the initial campaign, the TikTok campaign achieved more impressions than the Meta campaign, slightly more budget (€257.18) than predetermined (€200) was used for the TikTok channel during the boost campaign. There were 201,689 impressions and 1,153 clicks per landing page view. The budget spent on the boost campaign on Meta was €83.37. The reach was 12,180 unique individuals, with 30,388 impressions, 157 clicks per landing page view, and a cost per click of €0.53.

For the website, there were 1,831 page views over the period from December 14 th, 2022, to January 20 th, 2023, out of which 1,749 were unique.

An email requesting students to complete the post-intervention questionnaire was sent out to all students of the University of Antwerp and all students of Ghent University. Of these students, 7.63% (1,827/23,944) from the University of Antwerp completed the post-intervention questionnaire. The response rate at Ghent University was 6.51% (3,334/51,237).

Among the students of the University of Antwerp who completed the process evaluation questions, 18.67% (225/1205) of the respondents were exposed to the social media SNA campaign; 22.3% (165/740) of the female students and 12.9% (60/464) of the male students. Among them, 64.0% (144/225) reported having seen the campaign via Facebook, 53.3% (120/225) via Instagram, 3.1% (7/225) via TikTok, and 11.6% (26/225) via other channels (student portal, website, university). One third of the students (75/225, 33.3%) saw the campaign through multiple channels.

Among the 225 exposed survey participants, 60.4% (136/225) were exposed to image 1 (Fig. 1a), 44.9% (101/225) to image 2 (Fig. 1b), 35.1% (79/225) to image 3 (Fig. 1c), 13.8% (31/225) to video 1 (Fig. 1d), 10.7% (24/225) to video 2 (Fig. 1e), and 30.2% (68/225) to the website. Some students were exposed to more than one component (images, videos, and/or website) of the campaign: 23.1% (52/225) students were exposed to two components, 21.8% (49/225) students to three components, 4.0% (9/225) to four components, 1.3% (3/225) to five components, and 2.2% (5/225) to all components of the campaign.

83.6% (179/214) of the exposed students found the campaign credible, and about half of the students (48.6%, 104/214) considered the campaign as appealing; 54.9% (90/164) among the female students and 40.0% (24/60) of the male students. Most of the students reported that the website, videos, and images were clear (respectively 90.8% (59/65), 94.7% (36/38), and 92.4% (158/171)).

The characteristics of the students of the University of Antwerp who were exposed to the SNA intervention and those who were not, were similar, except for sex; 73.3% (165/225) of the students who reported having seen the campaign were female, however, among the students who reported not having seen the campaign, only 58.7% (575/980) were female [see Additional file 7].

#### Mechanisms of impact

The mean score of the overall perceived level of satisfaction about the intervention of the 216 respondents was 6.4 (SD 1.7), and the mode and the median were both 7. For the static images, the mean perceived satisfaction (*n* = 176) was 6.6 (SD 1.7), and the median and the mode were 7, for the videos (*n* = 38), a mean score of 5.3 (SD 1.7), mode 6 and median 6, and for the website (*n* = 65) a mean score of 5.8 (SD 1.7), and mode 8 and median 6.

The majority of the students who were exposed to the intervention perceived benefits of the intervention: 62.8% (135/215) of the students agreed that the campaign has made them aware that students are less likely to use stimulant medication to improve their study performance than they thought. One out of twelve (8.3%, 1/12) users of prescription stimulants for study performance were encouraged to use less stimulant medication as a result of the campaign, and two out of twelve (16.7%, 2/12) students using prescription stimulants to enhance their study performance considered that the campaign made them take a different view on their own use of stimulant medication to improve their study performance.

In contrast as a result of the campaign, 16.3% (35/215) of the students were aware that students use stimulant medication to improve their study performance more often than they thought. Furthermore, a small group of students (1.9%, 4/215) reported being encouraged to use more stimulant medication as a result of the campaign.

The students support campaigns on NMUPS; 88.4% (191/216) of the students stated that it is important that these kinds of campaigns on stimulant medication are done, and just 15.7% (34/216) indicated that too much attention is paid to the subject of stimulant medication in students.

The campaign made 43.3% (90/208) of the students think about students’ use of prescription stimulants to improve study performance, and two out of nine (22.2%, 2/9) stimulant users have thought about their own use of stimulants to improve their study performance. 5.8% (12/208) talked about the use of prescription stimulants by themselves or by students in their environment. However, almost nobody (0.5%, 1/207) had sought help as a result of the campaign.

#### Context

The context might play a significant role in shaping the campaign’s implementation. For instance, the campaign material had to fit with the University of Antwerp's corporate identity. This entailed some restrictions, such as that images were not allowed to contain text, and messages had to be placed above and below the images. One student commented that the photos alone did not convey a message and required reading the caption to understand them.

Besides, the effect of exposure to other campaigns during the study period should be considered. However, at endpoint, only 0.25% (3/1,186) of the students of the University of Antwerp and 0.35% (7/1,989) of the students of Ghent University reported being exposed to other campaigns on NMUPS for study performance.

## Discussion

Results of the study indicate that the SNA intervention had a significant effect on students'perception of NMUPS for study performance, aligning it more closely with the actual social norm. However, no significant effect on the actual usage was demonstrated. Caution should be offered in making causal inferences because only measured confounders were taken into account. Nevertheless, these confounders were equally distributed between the intervention and control groups using IPW.

A possible reason for not observing an effect on NMUPS could be that the primary outcome variable was past-year NMUPS for study enhancement, and the campaign was launched less than 12 months before the endline measurement. The HITC ed. 2021 survey only assessed past-year NMUPS for study enhancement. For consistency and to facilitate comparison between baseline and endline data, the same question was repeated in the post-intervention survey. This approach may have underestimated the effect of the SNA campaign on the NMUPS for study enhancement. Moreover, the number of students using NMUPS for study performance who had both seen the SNA campaign and completed the post-intervention questionnaire was limited.

The finding that there was no statistically significant effect of the SNA intervention on NMUPS for study performance is in line with the results of a systematic review of alcohol misuse in students. This review concluded that SNA interventions in the drug and alcohol field are often found to be ineffective or have low effectiveness. Although, the authors suggested that the SNA could still be valuable as part of a broader prevention campaign [51].

In our study, a notable increase in the NMUPS for study performance was observed in both the intervention and control groups at the final measurement. Although caution should be used, a possible explanation for the lower NMUPS at baseline (2021) could be the COVID- 19 pandemic. In 2021, 4.4% of Flemish students reported the use of prescribed stimulants to improve their study performance, while in 2017 the percentage was 6.5% [5, 52].

This study used a self-reported questionnaire to obtain information on NMUPS for study performance. This may have resulted in socially desirable responses from some students. To mitigate this, it was clearly stated that questionnaires were completed anonymously. Nevertheless, we must take into account the possible social desirability bias of self-reported outcomes. Reliance on self-reporting may also introduce recall bias.

However, the influence of the COVID- 19 pandemic and self-reported outcomes should be minimal because of the pre-post design with a control group. This design helps isolate intervention effects by comparing changes within groups over time. Still, the results should be interpreted with caution because not the same students participated in the pre- and post-measurement. This may have led to selection bias, especially if students with different characteristics completed the questionnaires. In any case, due to the convenience sampling method, selection bias may have occurred if certain groups of students felt more inclined to complete the questionnaires. This could have resulted in a non-representative depiction of the overall student population.

The campaign consisted of social norm messages which were based on data of the students of the University of Antwerp from the HITC survey ed. 2021. This data was also obtained using convenience sampling, and, in addition, no weighting was applied. Therefore, these results, and thus the social norm messages, may not be fully representative of the broader Antwerp student population.

Also regarding the campaign, it was surprising that the more reach certain components had, the fewer students reported seeing them. Additionally, during the boost campaign, TikTok was found to be more successful than Meta, although this was not reflected in the responses to the post-intervention questionnaire. This discrepancy could suggest that students of the University of Antwerp who were the target audience of the TikTok campaign either did not complete the post-intervention questionnaire, leading to potential selection bias, or – more plausibly—that the TikTok campaign did not effectively reach the students of the University of Antwerp. Since 35.7% of the total campaign budget (€457/€1280) had been spent on TikTok, we may conclude that the campaign budget could have been used more optimally to reach a maximal intervention implementation. Additionally, another limitation of the campaign is the lack of clarity regarding the ideal duration for an SNA campaign.

Given the inherent unpredictability of social media campaigns, it is challenging to determine in advance whether a campaign will succeed or fail. Some posts go viral, while others receive little engagement. Consequently, replicating social media interventions in future studies is almost impossible.

Nevertheless, our research is important because it is one of the few studies that addressed common pitfalls of SNA research; by using a pre-intervention baseline measurement from the target group, including a comparison group, performing a process evaluation, and the use of clear reporting according to the TREND guidelines. Other challenges that had been faced were the dissemination of the campaign through innovative and appealing channels for the target group, such as social media. This approach may have benefited the external validity of the study.

Further follow-up research will have to show whether there will be a significant effect on NMUPS for study performance in the longer term. This might suggest that there was a lack of time for the intervention to take effect on the students’ behavior. The long-term maintenance of the intervention should be assessed by examining the sustainability of awareness change and possible behavior change among the students. To maximize the effect, close involvement of the target audience should be aimed for in the development of campaigns. Additionally, incorporating qualitative research alongside quantitative methods could provide valuable insights into the facilitators and barriers related to the implementation of the intervention. Further attention should also focus on clarifying the ideal duration of an SNA campaign, as well as understanding differences in responses to the intervention between sexes. Finally, future research will need to show whether the results can be generalized to other university populations.

## Supplementary Information

Additional file 1. TREND Statement Checklist

Additional file 2. Process evaluation: statements and questions for the different parts of the campaign and the campaign as a whole.

Additional file 3. DAGs of relationship between SNA intervention and (perception of) NMUPS

Additional file 4. Distribution of data missingness at baseline and endline

Additional file 5. Inverse Probability Weighting

Additional file 6. Exploratory post-hoc subgroup analysis within male and female students

Additional file 7. Characteristics of students of University of Antwerp who have/have not seen the SNA campaign

## Data Availability

The datasets used and/ or analyzed are available from the corresponding author upon reasonable request.

## Abbreviations

ADHD: Attention deficit hyperactivity disorder
AUDIT-C: Alcohol use disorders identification test – consumption
CSSS: College student stress scale
DAG: Directed acyclic graph
DiD: Difference in difference
GLMM: Generalized linear mixed model
HITC: Heads in the clouds
IPW: Inverse probability weighting
MAR: Missing at random
MI: Multiple imputation
MRC: Medical Research Council
NMUPS: Nonmedical use of prescription stimulants
PSM: Propensity score matching
SAPS: Short revised almost perfect scale
SNA: Social norms approach
TREND: Transparent reporting of evaluations with nonrandomized designs
US: United States
VAD: Flemish expertise center on alcohol and other drugs
