# Supplementary material for: Outcome and process evaluation of a social norms approach intervention on nonmedical use of prescription stimulants for study performance among Flemish university students: a quasi-experimental study
Source: Arch Public Health. 2025 Jun 6;83:145. doi: 10.1186/s13690-025-01603-6 (PMC12142950; doi:10.1186/s13690-025-01603-6)
Supplement: Supplementary file 2 — Additional file 2. Process evaluation: statements and questions for the different parts of the campaign and the campaign as a whole. [file 13690_2025_1603_MOESM2_ESM.pdf]

Additional file 2: Process evaluation: statements and questions (in Dutch)

- for the different parts of the campaign (images, videos, and website)

Example concerns process evaluation questions related to the images. Questions about videos and website were similar.

In hoeverre ben je het eens met de volgende stellingen over **deze afbeeldingen/posts**?

Kies het toepasselijke antwoord voor elke stelling:

	Helemaal oneens	Oneens	Eens	Helemaal eens
Ik vind de posts duidelijk.	○	○	○	○
De posts hebben mij aangezet om meer stimulerende medicatie te gebruiken om mijn studieprestaties te verbeteren.	○	○	○	○
De posts hebben mij aangezet om minder stimulerende medicatie te gebruiken om mijn studieprestaties te verbeteren.	○	○	○	○
De posts hebben mij ervan bewust gemaakt dat studenten minder vaak stimulerende medicatie gebruiken om hun studieprestaties te verbeteren dan ik dacht.	○	○	○	○
De posts hebben mij ervan bewust gemaakt dat studenten vaker stimulerende medicatie gebruiken om hun studieprestaties te verbeteren dan ik dacht.	○	○	○	○

Wat vond je van **<u>de afbeeldingen/posts</u>** op een schaal van 0 tot 10?

Waarbij 0 = zeer slecht en 10 = zeer goed

0 1 2 3 4 5 6 7 8 9 10

- for the campaign as a whole

In hoeverre ben je het eens met de volgende stellingen over de inhoud van deze studentencampagne over stimulerende medicatie?

Kies het toepasselijke antwoord voor elk onderdeel:

	Helemaal oneens	Oneens	Eens	Helemaal eens
Er wordt te veel aandacht besteed aan het onderwerp stimulerende medicatie bij studenten.	○	○	○	○
Het is belangrijk dat dit soort campagnes over stimulerende medicatie gedaan worden.	○	○	○	○
De campagne spreekt mij aan.	○	○	○	○
De campagne is geloofwaardig.	○	○	○	○
Door deze campagne ben ik mij ervan bewust geworden dat studenten minder vaak stimulerende medicatie gebruiken om hun studieprestaties te verbeteren dan ik dacht.	○	○	○	○
Door deze campagne ben ik mij ervan bewust geworden dat studenten vaker stimulerende medicatie gebruiken om hun studieprestaties te verbeteren dan ik dacht.	○	○	○	○
De campagne zette me aan om minder stimulerende medicatie te gebruiken om mijn studieprestaties te verbeteren.	○	○	○	○
De campagne zette me aan om meer stimulerende medicatie te gebruiken om mijn studieprestaties te verbeteren.	○	○	○	○
Door de campagne heb ik een andere visie gekregen op mijn eigen gebruik van stimulerende medicatie om mijn studieprestaties te verbeteren.	○	○	○	○

Geef een cijfer van 0 tot 10 waarbij 0 = zeer slecht en 10 = zeer goed

0	1	2	3	4	5	6	7	8	9	10

Heb je naar aanleiding van de campagne...

	Ja	Nee
...contact opgenomen met of hulp gezocht bij ...?	○	○
...nagedacht over je eigen gebruik van stimulerende middelen om je studieprestaties te verbeteren?	○	○
...nagedacht over het gebruik van stimulerende middelen van studenten om studieprestaties te verbeteren?	○	○
...gepraat over het gebruik van stimulerende middelen om studieprestaties te verbeteren van uzelf of van mensen uit uw omgeving?	○	○
