# Supplementary figures and images for: Outcome and process evaluation of a social norms approach intervention on nonmedical use of prescription stimulants for study performance among Flemish university students: a quasi-experimental study
Source: Arch Public Health. 2025 Jun 6;83:145. doi: 10.1186/s13690-025-01603-6 (PMC12142950; doi:10.1186/s13690-025-01603-6)

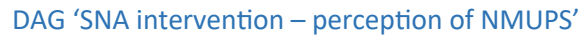

Supplement: Supplementary file 3 — Additional file 3. DAGs of relationship between SNA intervention and (perception of) NMUPS [file 13690_2025_1603_MOESM3_ESM.pdf]
