# Supplementary material for: Outcome and process evaluation of a social norms approach intervention on nonmedical use of prescription stimulants for study performance among Flemish university students: a quasi-experimental study
Source: Arch Public Health. 2025 Jun 6;83:145. doi: 10.1186/s13690-025-01603-6 (PMC12142950; doi:10.1186/s13690-025-01603-6)
Supplement: Supplementary file 4 — Additional file 4. Distribution of data missingness at baseline and endline [file 13690_2025_1603_MOESM4_ESM.pdf]

Additional file 4: Distribution of data missingness at baseline and endline

Distribution of missingness at baseline

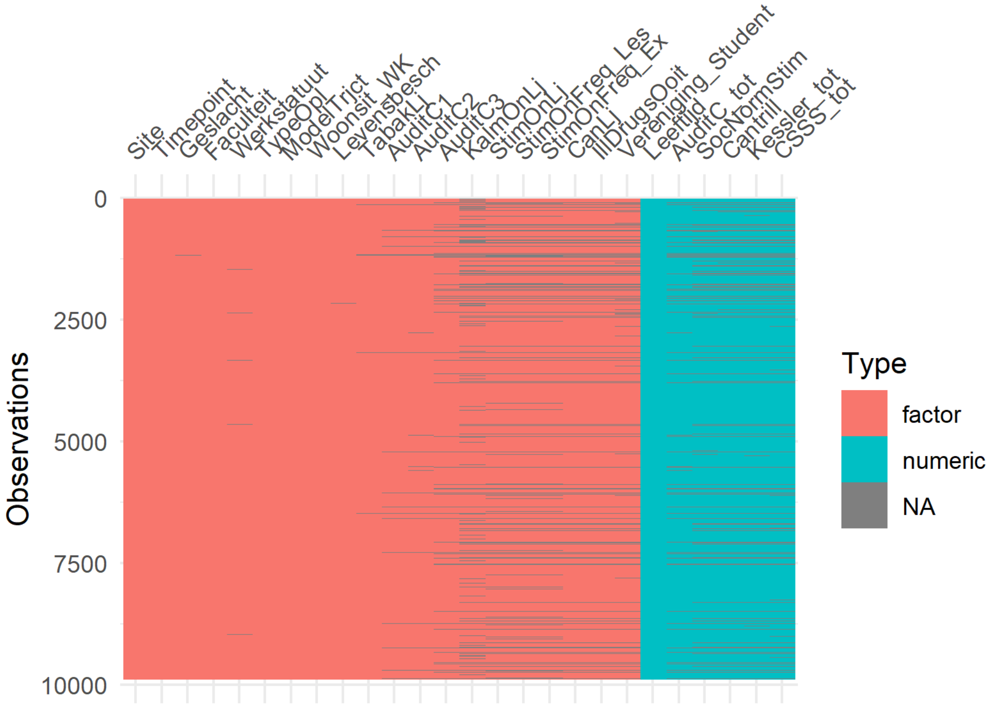

variable <chr>	n_miss <int>	pct_miss <dbl>
KalmOnLj	1931	19.53662485
StimOnFreq_Les	1457	14.74099555
StimOnFreq_Ex	1455	14.72076083
Vereniging_Student	1455	14.72076083
StimOnLj	1447	14.63982193
CSSS_tot	1426	14.42735735
Kessler_tot	1352	13.67867260
Cantrill	1319	13.34479968
IllDrugsOoit	1274	12.88951841
SocNormStim	1270	12.84904897
CanLj	1260	12.74787535
AuditC_tot	772	7.81060299
AuditC3	732	7.40590854
AuditC2	235	2.37757993
AuditC1	193	1.95265075
Werkstatuut	81	0.81950627
TabakLj	75	0.75880210
ModelTrjct	23	0.23269931
Faculteit	21	0.21246459
Levensbesch	10	0.10117361
Woonsit_WK	4	0.04046945
Geslacht	3	0.03035208
Site	0	0.00000000
Timepoint	0	0.00000000
Leeftijd	0	0.00000000
TypeOpl	0	0.00000000

Distribution of missingness at endline

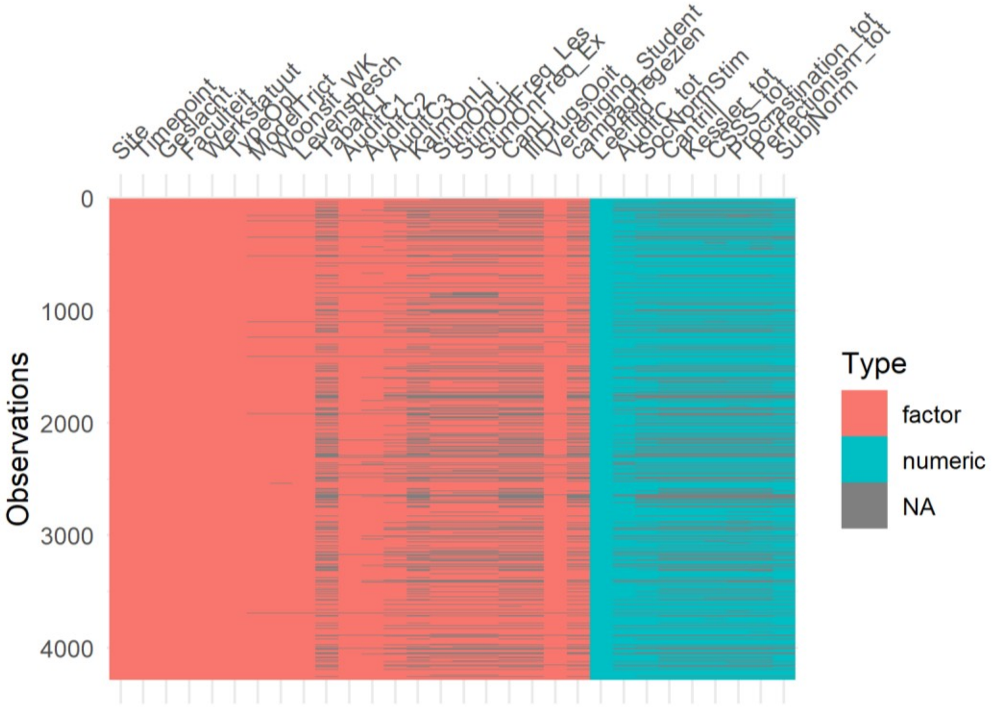

variable <chr>	n_miss <int>	pct_miss <dbl>
Procrastination_tot	1398	32.66355140
Perfectionism_tot	1362	31.82242991
CSSS_tot	1335	31.19158879
Kessler_tot	1321	30.86448598
CanLj	1320	30.84112150
TabakLj	1319	30.81775701
KalmOnLj	1318	30.79439252
IllDrugsOoit	1316	30.74766355
Cantrill	1307	30.53738318
StimOnFreq_Les	1195	27.92056075
StimOnFreq_Ex	1194	27.89719626
StimOnLj	1119	26.14485981
SubjNorm	1091	25.49065421
SocNormStim	1086	25.37383178
campagnegezien	1080	25.23364486
AuditC_tot	685	16.00467290
AuditC3	666	15.56074766
AuditC2	214	5.00000000
AuditC1	144	3.36448598
Vereniging_Student	140	3.27102804
ModelTrjct	75	1.75233645
Woonsit_WK	72	1.68224299
Levensbesch	64	1.49532710
Werkstatuut	9	0.21028037
Geslacht	3	0.07009346
TypeOpl	3	0.07009346
Faculteit	2	0.04672897
Site	0	0.00000000
Timepoint	0	0.00000000
Leeftijd	0	0.00000000
