# Supplementary material for: Outcome and process evaluation of a social norms approach intervention on nonmedical use of prescription stimulants for study performance among Flemish university students: a quasi-experimental study
Source: Arch Public Health. 2025 Jun 6;83:145. doi: 10.1186/s13690-025-01603-6 (PMC12142950; doi:10.1186/s13690-025-01603-6)
Supplement: Supplementary file 5 — Additional file 5. Inverse Probability Weighting [file 13690_2025_1603_MOESM5_ESM.pdf]

Additional file 5: Inverse Probability Weighting

Below plots are based on the combined dataset of all multiple imputed datasets (m=10). These plots were made for all separate datasets and assumptions were checked for all the different imputed datasets.

Plot of propensity scores for baseline and endline data

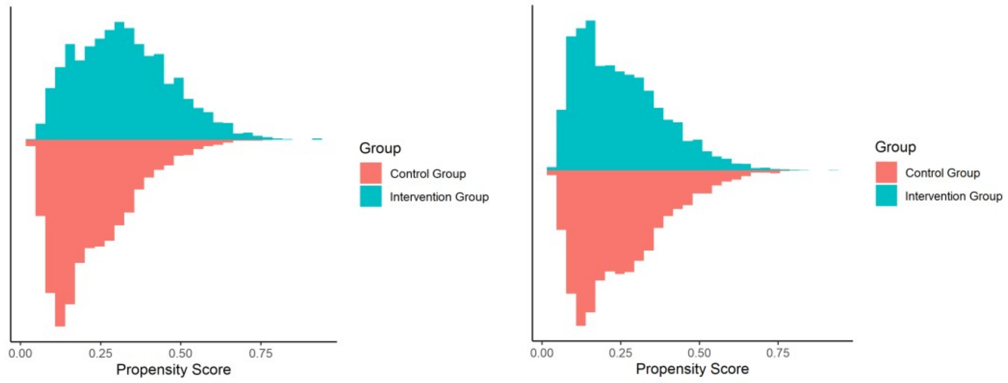

Figure a. Plot of propensity scores for baseline data

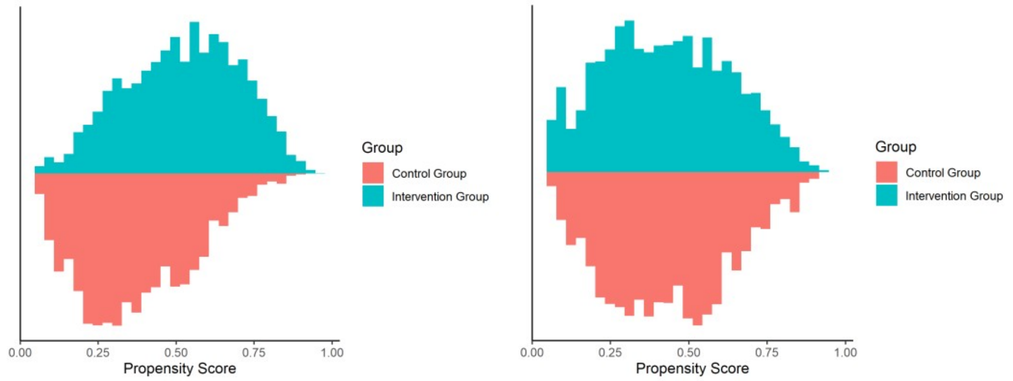

Figure b. Plot of propensity scores for endline data

Assumptions of IPW

(1) (Conditional) exchangeability

Intervention and control groups are exchangeable (within each stratum of the confounding variables).

	Stratified by intervention 0	1	SMD
n	98893.3	98725.0	
Geslacht = 2 (%)	37487.7 (37.9)	36598.9 (37.1)	0.017
Leeftijd (mean (SD))	20.80 (1.97)	20.89 (1.97)	0.049
Werkstatuut (%)			0.026
1	73986.0 (74.8)	73384.0 (74.3)	
2	21481.3 (21.7)	21429.8 (21.7)	
3	3425.9 (3.5)	3911.2 (4.0)	
Faculteit (%)			0.022
6	15992.5 (16.2)	16024.1 (16.2)	
5	12298.7 (12.4)	12245.1 (12.4)	
2	8648.9 (8.7)	8586.9 (8.7)	
1	16717.6 (16.9)	16733.8 (16.9)	
7	11261.2 (11.4)	11834.0 (12.0)	
3	18345.9 (18.6)	17749.7 (18.0)	
8	7736.4 (7.8)	7694.2 (7.8)	
4	7892.0 (8.0)	7857.2 (8.0)	
TypeOpl (%)			0.059
2	56903.4 (57.5)	54973.0 (55.7)	
4	2945.1 (3.0)	3225.8 (3.3)	
1	5434.6 (5.5)	6663.6 (6.7)	
3	33610.2 (34.0)	33862.5 (34.3)	

ModelTrjct = 2 (%)	31641.9 (32.0)	31402.2 (31.8)	0.004
Woonsit_WK = 2 (%)	53353.2 (54.0)	53512.3 (54.2)	0.005
Levensbesch (%)			0.019
1	35671.9 (36.1)	36075.1 (36.5)	
2	97.9 (0.1)	117.1 (0.1)	
3	2360.5 (2.4)	2306.4 (2.3)	
4	170.2 (0.2)	194.5 (0.2)	
5	372.5 (0.4)	444.3 (0.5)	
6	56884.1 (57.5)	56169.3 (56.9)	
7	3336.1 (3.4)	3418.3 (3.5)	
TabakLj = 2 (%)	74873.0 (75.7)	75477.3 (76.5)	0.017
AuditC_tot (mean (SD))	6.07 (2.40)	6.02 (2.57)	0.023
KalmOnLj = 2 (%)	95803.0 (96.9)	95612.3 (96.8)	0.002
CanLj = 2 (%)	71775.8 (72.6)	72485.3 (73.4)	0.019
IllDrugsOoit = 2 (%)	85392.2 (86.3)	85369.2 (86.5)	0.004
Cantrill (mean (SD))	6.10 (1.71)	6.07 (1.73)	0.015
Kessler_tot (mean (SD))	17.38 (5.23)	17.48 (5.28)	0.020
CSSS_tot (mean (SD))	29.78 (7.89)	29.94 (7.90)	0.020
Vereniging_Student = 1 (%)	13018.2 (13.2)	12553.4 (12.7)	0.013

Table 1. Standardized mean differences of measured and selected confounders between control and intervention group at baseline after weighting

	Stratified by intervention		
	0	1	SMD
n	35200.2	35902.5	
Geslacht = 2 (%)	13394.4 (38.1)	13936.6 (38.8)	0.016
Leeftijd (mean (SD))	20.71 (1.92)	20.77 (2.15)	0.029
Werkstatuut (%)			0.012
1	22894.4 (65.0)	23546.8 (65.6)	
2	11120.1 (31.6)	11134.1 (31.0)	
3	1185.6 (3.4)	1221.6 (3.4)	
Faculteit (%)			0.038
1	6057.7 (17.2)	5999.2 (16.7)	
2	3588.5 (10.2)	3672.0 (10.2)	
3	5389.0 (15.3)	5366.0 (14.9)	
4	3803.7 (10.8)	3759.9 (10.5)	
5	3313.9 (9.4)	3330.9 (9.3)	
6	5237.2 (14.9)	5805.9 (16.2)	
7	5251.2 (14.9)	5393.6 (15.0)	
8	2558.9 (7.3)	2575.0 (7.2)	
TypeOpl (%)			0.057
2	22124.6 (62.9)	22231.2 (61.9)	
4	758.6 (2.2)	1093.2 (3.0)	
1	1803.9 (5.1)	1805.3 (5.0)	
3	10513.1 (29.9)	10772.9 (30.0)	
ModelTrjct = 2 (%)	11681.5 (33.2)	12047.6 (33.6)	0.008
Woonsit_WK = 2 (%)	18524.8 (52.6)	19042.6 (53.0)	0.008
Levensbesch (%)			0.010
1	9782.2 (27.8)	9946.6 (27.7)	
2	49.9 (0.1)	47.1 (0.1)	
3	775.7 (2.2)	791.0 (2.2)	
4	41.8 (0.1)	34.0 (0.1)	
5	91.4 (0.3)	87.3 (0.2)	
6	23143.5 (65.7)	23627.9 (65.8)	
7	1315.8 (3.7)	1368.7 (3.8)	
TabakLj = 2 (%)	23906.5 (67.9)	24499.1 (68.2)	0.007
AuditC_tot (mean (SD))	6.50 (2.92)	6.51 (3.01)	0.003
KalmOnLj = 2 (%)	32628.3 (92.7)	33296.4 (92.7)	0.002
CanLj = 2 (%)	25898.6 (73.6)	26347.6 (73.4)	0.004
IllDrugsOoit = 2 (%)	29861.1 (84.8)	30510.4 (85.0)	0.004
Cantrill (mean (SD))	6.86 (1.43)	6.84 (1.43)	0.013
Kessler_tot (mean (SD))	15.77 (4.72)	15.77 (4.57)	<0.001
CSSS_tot (mean (SD))	29.20 (7.70)	29.18 (7.18)	0.003
Vereniging_Student = 1 (%)	6569.3 (18.7)	6653.3 (18.5)	0.003
Procrastination_tot (mean (SD))	39.66 (5.78)	39.70 (5.59)	0.008
Perfectionism_tot (mean (SD))	37.07 (8.61)	37.09 (8.19)	0.003
SubjNorm (mean (SD))	3.57 (1.63)	3.59 (1.60)	0.013

Table 2. Standardized mean differences of measured and selected confounders between control and intervention group at endline after weighting

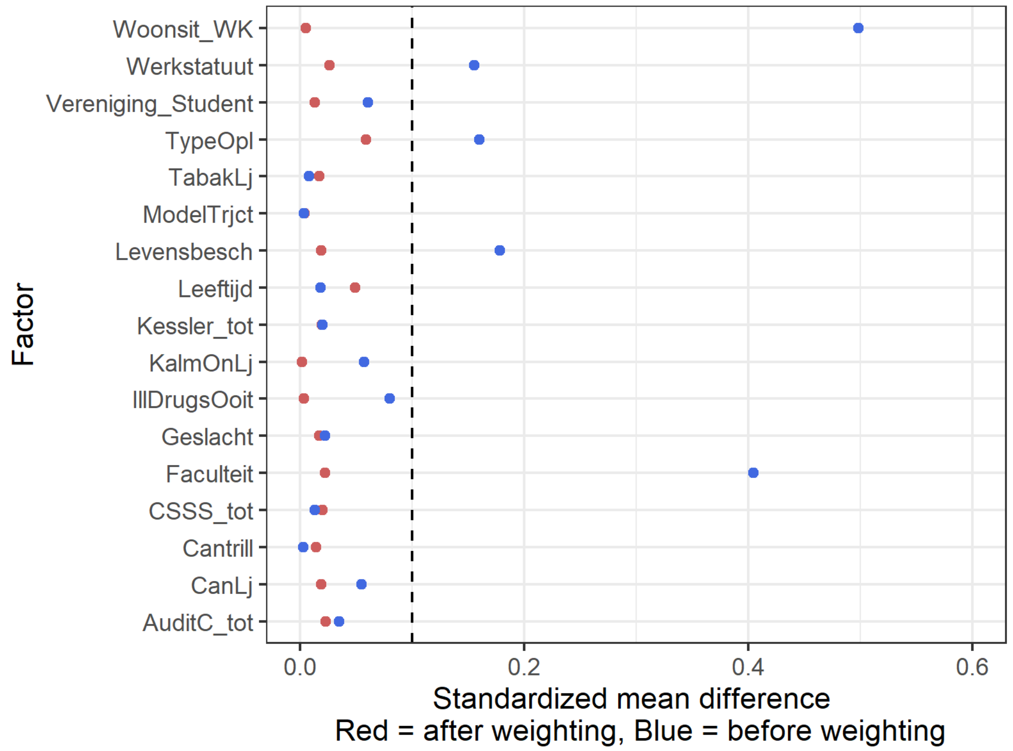

Figure c. Comparison SMD before and after weighing for baseline data.

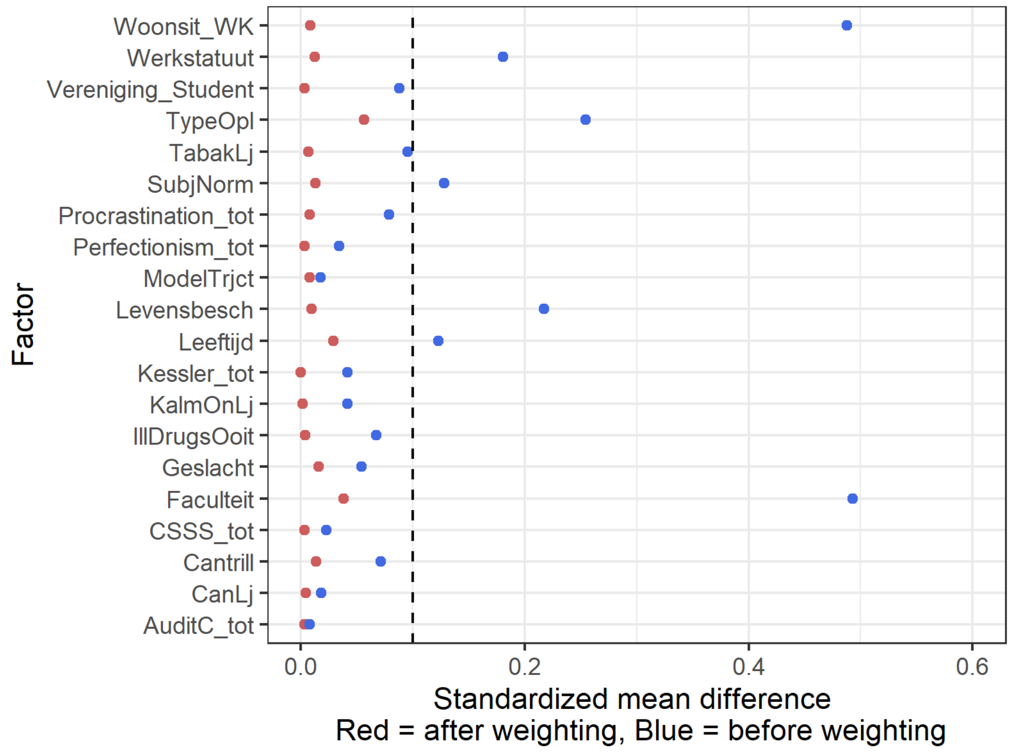

Figure d. Comparison SMD before and after weighing for baseline data.

(2) Positivity

Tables 1 and 2 show that there are for each level of every confounder exposed and unexposed students.

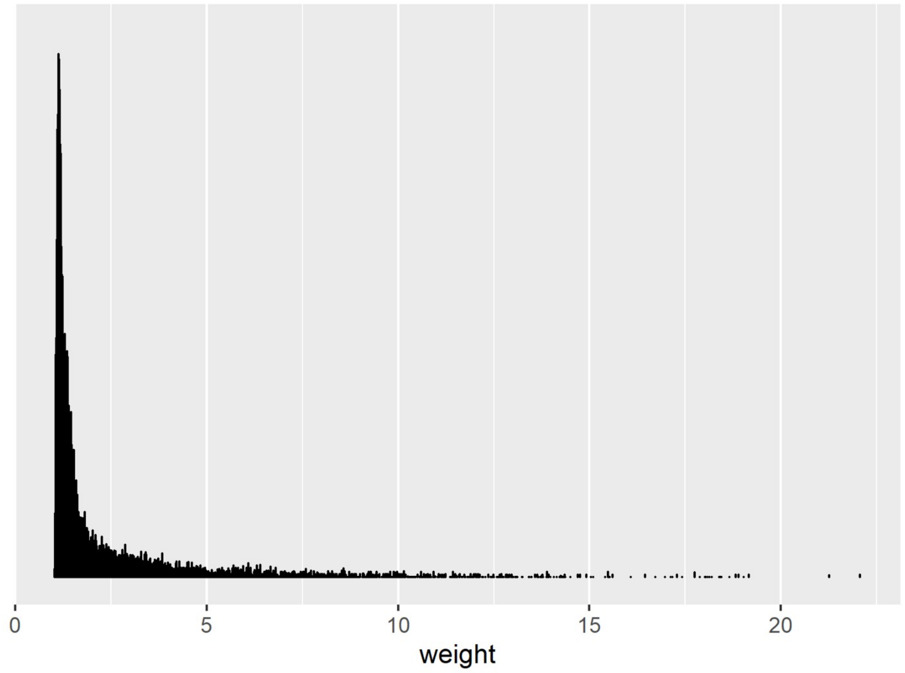

Figure e. Weights vary between 1.026 and 22.062 for baseline.

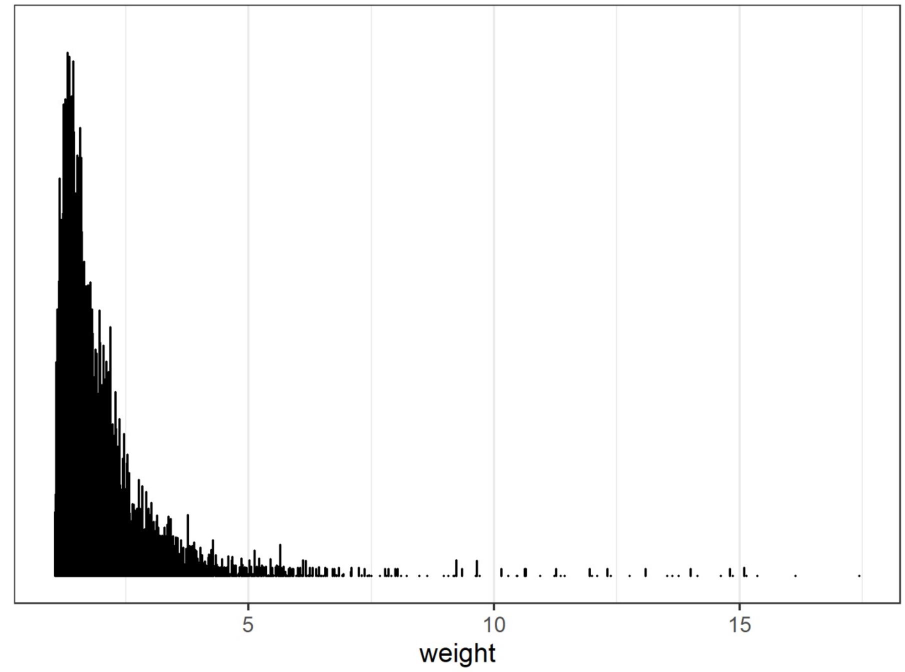

Figure f. Weights vary between 1.051 and 17.444 for endline.

(3) Consistency

The intervention is well-defined and any variation will not have a different impact on the outcome.
