# Supplementary material for: Outcome and process evaluation of a social norms approach intervention on nonmedical use of prescription stimulants for study performance among Flemish university students: a quasi-experimental study
Source: Arch Public Health. 2025 Jun 6;83:145. doi: 10.1186/s13690-025-01603-6 (PMC12142950; doi:10.1186/s13690-025-01603-6)
Supplement: Supplementary file 6 — Additional file 6. Exploratory post-hoc subgroup analysis within male and female students [file 13690_2025_1603_MOESM6_ESM.pdf]

Additional file 6: Exploratory post-hoc subgroup analysis within male and female students

Table a. Effect of intervention within subgroup of male students on perceived social norm of NMUPS for study performance.

Fixed effects			
	Est.	SE	*p*
Intercept	32.337	0.668	< .0001
Time			
Endline	1.554	0.884	0.079
Group			
intervention	0.206	0.687	0.765
Time*group			
Endline*intervention	-2.992	1.250	0.0167

Random effects			
	Est.	Std. dev	N group
Faculty (intercept)	$1.030\ 10^{-13}$	1.031	8

Table b. Effect of intervention within subgroup of female students on perceived social norm of NMUPS for study performance.

Fixed effects			
	Est.	SE	*p*
Intercept	38.882	0.732	< .0001
Time			
Endline	-0.684	0.706	0.333
Group			
intervention	0.132	0.557	0.813
Time*group			
Endline*intervention	-4.196	1.017	< .0001

Random effects			
	Est.	Std. dev	N group
Faculty (intercept)	$5.032\ 10^{-13}$	1.649	8
