# Supplementary material for: Outcome and process evaluation of a social norms approach intervention on nonmedical use of prescription stimulants for study performance among Flemish university students: a quasi-experimental study
Source: Arch Public Health. 2025 Jun 6;83:145. doi: 10.1186/s13690-025-01603-6 (PMC12142950; doi:10.1186/s13690-025-01603-6)
Supplement: Supplementary file 7 — Additional file 7. Characteristics of students of University of Antwerp who have/have not seen the SNA campaign [file 13690_2025_1603_MOESM7_ESM.pdf]

Additional file 7: Characteristics of the students of University of Antwerp who have/ have not seen the SNA campaign

Table a. Students' characteristics of intervention group at endline: those who have seen the SNA campaign versus those who have not seen the SNA campaign.

		Intervention group at Endline n=1,544	
		**Seen** *n=225*	**Not seen** *n=980*
Sex, n (%)	Female	165 (73.3)	575 (58.7)
	Male	60 (26.7)	404 (41.3)
Age (years), median [IQR]		21.0 [3.0]	20.0 [3.0]
Faculty, n (%)	Medicine and health sci.	37 (16.4)	160 (16.3)
	Veterinary and pharm. sci.	38 (16.9)	165 (16.8)
	Engineering sciences	19 (8.4)	145 (14.8)
	Exact sciences	26 (11.6)	143 (14.6)
	Economics	27 (12.0)	83 (8.5)
	Social sci. and psychology	25 (11.1)	74 (7.6)
	Linguistics and philosophy	30 (13.3)	138 (14.1)
	Law and Criminology	23 (10.2)	72 (7.4)
Type of education, n (%)	Bridging program	13 (5.8)	54 (5.5)
	Bachelor's program	151 (67.1)	671 (68.5)
	Master's program	59 (26.2)	243 (24.8)
	Other	2 (0.9)	12 (1.2)
Living situation during week, n (%)	At parental home	142 (63.1)	601 (61.5)
	Independently	83 (36.9)	376 (38.5)
Working status, n (%)	Not working	130 (57.8)	588 (60.1)
	< 20 hours / week	84 (37.3)	351 (35.9)
	> 20 hours / week	11 (4.9)	39 (4.9)
Religion, n (%)	Christian	57 (25.3)	249 (25.4)
	Jewish	0 (0.0)	1 (0.10)
	Islamic	12 (5.3)	26 (2.7)
	Hindu	0 (0.0)	2 (0.2)
	Buddhist	0 (0.0)	1 (0.1)
	No religion	152 (67.6)	654 (66.7)
	Other	4 (1.8)	47 (4.8)
Active member student organization, n (%)	Yes	53 (23.6)	206 (21.1)
Social norm, mean (SD)		29.8 (19.9)	34.2 (19.3)
Last-year-NMUPS,n (%)	Yes	18 (8.6)	83 (9.1)
Last-year-tobacco use, n (%)	Yes	57 (28.1)	305 (32.6)
AUDIT-C score, median [IQR]		2.0 (5.0)	3.0 [5.0]
Last-year-tranquilizer use, n (%)	Yes	18 (9.0)	68 (7.3)
Last-year-cannabis use, n, (%)	Yes	47 (23.4)	243 (25.9)
Ever-use illegal drugs, n (%)	Yes	26 (12.9)	151 (16.1)
CSSS scale, median [IQR]		30.0 [11.8]	28.0 [11.0]
Kessler scale, median [IQR]		10.0 [6.0]	9.0 [7.0]
Cantril scale, median [IQR]		7.0 [2.0]	7.0 [2.0]
Perfectionism, median [IQR]		38.0 [12.0]	36.0 [12.0]
Procrastination, median [IQR]		40.0 [6.0]	40.0 [8.0]
